# A New 3D Mathematical Model for Simulating Nanofluid Flooding in a Porous Medium for Enhanced Oil Recovery

**DOI:** 10.3390/ma16155414

**Published:** 2023-08-02

**Authors:** Abdullah Al-Yaari, Dennis Ling Chuan Ching, Hamzah Sakidin, Mohana Sundaram Muthuvalu, Mudasar Zafar, Abdurrashid Haruna, Zulkifli Merican Aljunid Merican, Abdus Samad Azad

**Affiliations:** 1 Department of Fundamental and Applied Sciences, Universiti Teknologi PETRONAS, Seri Iskandar 32610, Malaysia; dennis.ling@utp.edu.my (D.L.C.C.); hamzah.sakidin@utp.edu.my (H.S.); mohana.muthuvalu@utp.edu.my (M.S.M.); mudasar_20000296@utp.edu.my (M.Z.); abdurrashid_21002269@utp.edu.my (A.H.); zulkifli.aljunid@utp.edu.my (Z.M.A.M.); abdus_19000955@utp.edu.my (A.S.A.); 2 Department of Mathematics, Faculty of Applied Science, Thamar University, Dhamar 00967, Yemen; 3 Department of Chemistry, Ahmadu Bello University, Zaria 810107, Nigeria

**Keywords:** mathematical model, EOR, nanofluid flooding, thermophysical properties, porous medium

## Abstract

Two-phase Darcy’s law is a well-known mathematical model used in the petrochemical industry. It predicts the fluid flow in reservoirs and can be used to optimize oil production using recent technology. Indeed, various models have been proposed for predicting oil recovery using injected nanofluids (NFs). Among them, numerical modeling is attracting the attention of scientists and engineers owing to its ability to modify the thermophysical properties of NFs such as density, viscosity, and thermal conductivity. Herein, a new model for simulating NF injection into a 3D porous media for enhanced oil recovery (EOR) is investigated. This model has been developed for its ability to predict oil recovery across a wide range of temperatures and volume fractions (VFs). For the first time, the model can examine the changes and effects of thermophysical properties on the EOR process based on empirical correlations depending on two variables, VF and inlet temperature. The governing equations obtained from Darcy’s law, mass conservation, concentration, and energy equations were numerically evaluated using a time-dependent finite-element method. The findings indicated that optimizing the temperature and VF could significantly improve the thermophysical properties of the EOR process. We observed that increasing the inlet temperature (353.15 K) and volume fraction (4%) resulted in better oil displacement, improved sweep efficiency, and enhanced mobility of the NF. The oil recovery decreased when the VF (>4%) and temperature exceeded 353.15 K. Remarkably, the optimal VF and inlet temperature for changing the thermophysical properties increased the oil production by 30%.

## 1. Introduction

Enhanced oil recovery methods are typically implemented to increase petroleum extraction from depleted oil reservoirs [1,2,3]. One of the methods used for EOR is nanofluid (NF) flooding, which involves the injection of a mixture of base fluid, such as water, and nanoparticles (NPs) into a reservoir to enhance oil mobility and recovery [4,5]. Nanofluids have attracted interest because of their distinctive characteristics and their ability to modify density, viscosity, and thermal conductivity with temperature and volume-fraction variations.

Over the past decade, numerous investigations have been carried out on the movement of NPs through PM [6,7]. These studies have explored how nanotechnology can be utilized in the oil industry to enhance oil recovery (OR). It has already been demonstrated that nanotechnology can be useful in the oil and gas sector, with NPs being suggested for various applications in the upstream oil industry such as NF flooding to help recover oil more effectively. There is a significant need to increase hydrocarbon energy production to meet growing energy demand. This problem can only be met by extracting as much as possible from the active reservoir, where a significant proportion of the oil remains, using EOR methods [8].

The initial phase of oil extraction, referred to as primary recovery, relies on the natural pressure within the reservoir to force oil to the surface. Over time, this pressure diminishes, resulting in a decline in oil production. Fewer than 30% of the reservoir’s oil is usually recovered during the first recovery. Following this, a secondary recovery phase is initiated, which often entails flooding the reservoir with water to increase OR by up to 50%. All extraction techniques utilized after primary and secondary recoveries are collectively known as EOR. According to the petroleum sector, the recovery factor in primary and secondary recovery methods ranges from 25% to 50%. Therefore, to meet the growing annual demand for oil, a tertiary recovery method such as chemical EOR is being implemented globally to obtain greater oil production from existing reservoirs [9].

Many recent experimental investigations have been conducted to evaluate the potential of NF flooding to increase OR. With the addition of NPs to water, oil recovery from porous media (PM) has significantly increased compared to conventional water flooding. These experiments commonly involve examining changes in properties such as viscosity, density, interfacial tension, wettability, and geometries to assess the effectiveness and efficiency of EOR. The focus of research on NFs in EOR has been on how various NPs influence these properties and the amount of oil that can be recovered from the PM. SiO${}_{2}$, TiO${}_{2}$, CuO, and SiO${}_{2}$ NPs are among the chemicals utilized in these experiments. To develop EOR techniques using NF flooding, it is necessary to understand the mechanism of NP interaction. Recent experimental research has shed light on how NFs, oil, and PM interact, which is a crucial aspect of the EOR process. More research is required to fully understand the EOR process via NF flooding [10].

Most NF flooding studies have been conducted experimentally. Still, researchers suggest modeling can be a more effective way to improve the EOR process and bring it closer to industrialization [11,12]. Models can supplement experiments and provide a detailed understanding of the EOR process. The addition of NPs to the base fluid for EOR has been researched numerically over the last decade by simulating oil displacement using mathematical models [13,14]. Using mathematical modeling to simulate oil displacement by NFs requires a computational grid to describe the structure of the PM and significant computational resources. Numerical studies can solve complex problems with fewer resources and accurately calculate modeling results. Two models, the mixing model and the immiscible fluid model, have been utilized to explain the multiphase flow of the base fluid and oil [15,16,17]. A model has been developed to simulate the NF using the concentration equation to control the movement of NPs through PM.

Several researchers have developed different approaches for developing a mathematical model to predict oil production after NFs are injected into oil reservoirs. Liu and Civan [12,18] proposed a prediction model for the oil recovery factor (ORF) by employing a two-phase flow of oil and water in sandstone. When NPs were added to the base fluid, the reservoir’s wettability and permeability changed. These changes were measured mathematically while considering pore-clogging and porosity alterations. The impacts of NP and pore surface relative permeabilities, capillary pressure, and wettability on two-phase flow were investigated. However, the drawback of their model is that it neglects the fluid density and viscosity changes caused by adding NPs to the fluid.

The deposition of NPs inside the pores of a reservoir can result in modifications to its permeability and porosity. Ju and Fan [19] enhanced a mathematical model that includes deriving equations for calculating changes in the reservoir’s porosity and permeability following the injection of NFs. This model could predict the OR after NF injection and evaluate the reservoir’s permeability and porosity. It was one of the first models developed to determine NP transportation in PM and has been frequently referenced by researchers in subsequent studies. This model was employed by Sepehri et al. [20] in a carbonate system, and its accuracy was confirmed by comparing it to experimental data. The results indicated that the model could predict a 10% enhancement in the ORF compared to the conventional water flooding method due to changes in wettability.

Ju and Fan [21] created a mathematical model that shows the movement of NPs in a two-phase flow through one-dimensional PM by adjusting the net rate of NP loss in their previous model. The model’s assumptions include (1) isothermal conditions with one-dimensional geometry; (2) incompressible PM and fluids; (3) oil and NFs flow through PM based on Darcy’s law, without considering gravity force; (4) NPs are divided into n-sized intervals; and (5) despite the addition of NPs, the viscosity and density of the fluids remain unchanged. NPs exist in either the oil or water phases based on their properties. Hydrophobic NPs are found in the oil phase, whereas hydrophilic NPs are found in the water phase. The transport of NFs through PM is facilitated by Brownian diffusion due to the wettability characteristics of the NFs. The authors improved the model to enhance surface deposition prediction and found that adding polysilicon NP to the base fluid improved OR [21].

El-Amin and colleagues modified the model developed by Ju and Fan by including a 2D displacement mechanism for EOR using non-Newtonian fluids. The study focused on the flow behavior of two-phase immiscible and incompressible fluids within a homogenous permeable material governed by Darcy’s law and the mass conservation equations for each phase. The authors developed a mathematical model to describe the absorption of NPs in a water–oil flow. The model incorporated capillary pressure and mixed relative permeability correlations for mixed-wet systems. The authors injected NFs into the oil reservoir to alter the wettability of the medium, causing it to change from oil-wet to water-wet. As a result, the rock was reclassified as a mixed-wet rock. During their research, they examined how NPs affected the properties of fluids and materials and conducted numerical studies to determine whether the OR process could be improved. However, the base fluid’s viscosity and density variations were not considered in their model [22].

Feng et al. extended the model developed by El-Amin et al. to investigate the impact of NP retention on oil displacement efficiency in a two-phase displacement and NP transport simulation study. The authors found that NF flooding following water flooding enhanced OR, and higher injection rates improved flooding efficiency. However, the study did not examine capillary pressure or changes in wettability [23]. Sepehri et al. [20] used the model developed by Darcy to examine how NPs affect wettability and cavitation generation in rock. In contrast, Yu et al. [24] discovered that water salinity significantly affects NP movement. Rahmani et al. [25] proposed a model to study the movement of superparamagnetic NPs within formations, aiming to assist in rock sounding using tracer diagnostics.

The loss of NPs in PM must be computed to predict their flow. Some researchers have investigated and computed the NP loss term using mechanical and empirical models. Gruesbeck and Collins [26] estimated NP deposition using an injection velocity-based expression to predict NP loss. Cullen et al. [27] proposed quantifying NP entrapment and modified the model to investigate how water saturation, NP concentration, porosity, and permeability affect the transport of NPs in PM. The improved model’s nonlinear governing equations were solved using the cell-centered finite-difference method.

Zhang et al. [28] developed a model to calculate NP concentrations at various time periods and core lengths. The concentration of NPs determined the amount of energy absorbed by the NF during injection into the core. Studies such as this one could provide insights into the optimal ways to combine electromagnetic heating and NP concentration. A limitation of the model was that it did not consider fluid saturation.

An et al. [29] developed a model that simulated the transport of NPs in reservoirs, considering both micro- and macro-scale phenomena. The model incorporated factors such as Darcy’s law, Brownian diffusion, gas diffusion, and capillary effects. The authors examined how oil and water flowed together in a two-phase system, where the water phase contained NPs and the oil phase contained methane. Although Darcy’s law governed the water flow, the oil flow was affected differently due to the relative permeability of the nanoscale pores. Since the NPs’ sizes were between 30 and 80 nm, the authors assumed that the flow was influenced by Brownian diffusion and convection. The authors made the following assumptions to examine NP mobility in shale reservoirs: NPs could only move in water; there was no NP concentration in the oil phase; and there was no interaction between NPs and the reservoir matrix.

Abdolfattah et al. [30] conducted a study on the mechanism of the wettability change in oil reservoirs during EOR using NPs based on the equations proposed by Liu and Sivan [18]. They developed a mathematical model to investigate how NPs adsorb in the reservoir, affecting wettability and changing the relative permeability of the two phases. In addition, Abdelfatah et al. [31] proposed a model combining Darcy’s equation with the convection-diffusion equation to analyze the movement, interaction, and behavior of NPs in the fluid, taking into account factors such as permeability, injection rate, formation volume, and NP size.

The research by Khalilinezhad et al. [32] explored the influence of adding NPs to fluids on their flow behavior in petroleum reservoirs. They employed a numerical method to validate their rheological findings and confirmed that NPs are adsorbed on sandstone surfaces. Their results revealed that NPs can reduce sandstone retention and adsorption. In a separate study, Gharibshahi et al. examined the influence of wettability on oil removal in a miscible model. They discussed the impact of the shape and distribution of PM in a two-dimensional microscopic model on the EOR process. They also discussed the roles of various factors in the oil-displacement process, including NP type, fluid velocity, particle size, and temperature of the injected fluid.

Furthermore, Salama et al. [33] developed a mathematical model to study the movement of NPs in PM. They used the filtration theory to explain the relationship between the mass deposition rate and the governing balance equation, resulting in the migration of fines and NP deposition. Esfe and Esfandeh [34] devised a model to explore how NFs can enhance OR in petroleum reservoirs. Their research investigated how the flow rate of NFs, the VF of nanoparticles, and the porosity of the PM affect EOR. The model was developed by adjusting the TPs of the injected NFs. The study concluded that increasing the flow rate of NFs positively impacted the EOR process, resulting in the recovery of more trapped oil. Additionally, an increase in the VF led to a change in the TPs of the base fluid.

In addition, some scientists found that for EOR, a consistent flow rate was more efficient in a low-porosity medium than in a high-porosity medium. Another study by Esfe et al. [35] created a new flooding model to simulate the flow of NFs in a petroleum reservoir filled with oil using two-phase Darcy’s law and mass transfer equations. The research examined the effects of mechanical parameters, such as fluid viscosity and density, on ORF. They also investigated the role of capillary pressure and permeability in the two-phase Darcy equations and found them to be critical parameters in the EOR process.

In their study, Al-Yaari et al. [36]. conducted a numerical simulation of NF flooding in PM, considering changes in thermophysical properties. Their results indicated that adding NPs at a VF ranging from 1% to 5% to a base fluid significantly increased ORF by over 25%. Furthermore, an increase in the inlet temperature resulted in EOR due to alterations in the viscosity and density of oil. The primary mechanism behind the EOR observed in the study was an increase in the relative permeability of the NF coupled with a simultaneous reduction in the relative permeability of the oil resulting from the presence of NPs.

Esfe et al. [37] developed a simulation model to replicate the EOR process by utilizing the two-phase Darcy equations. The model employed Raafat’s permeability model and Brooks–Corey’s capillary pressure model. The authors investigated the impact of the NF inlet temperature and VF on the OR rate. Although previous numerical studies have demonstrated the importance of modeling the oil-displacement process using NF injection to improve OR, only a few studies have addressed three-dimensional modeling. These models can predict how effective NFs will be in replacing oil using parameters such as NP mass fraction and size, displacement fluid flow rate, oil viscosity, and core permeability. The wetting properties of the medium have been the primary focus of previous models. Still, the change in TPs of the NF, such as density and viscosity, is also an important factor affecting the EOR process [34]. Although several models have been proposed to predict the ORF, no generalized model has been able to account for the density and viscosity of NFs across a wide range of temperatures and VFs.

Al-Yaari et al. [38] conducted a study to optimize the use of NPs in enhancing oil recovery in a 3D PM. The authors examined the effects of different types of NPs, their VFs, and the inlet temperature on oil recovery efficiency. The results showed that SiO${}_{2}$ NPs exhibited the best ORF compared to other NPs. Interestingly, SiO${}_{2}$ also had the lowest density and the highest thermal capacity among the examined NPs. The authors found that the optimum volume fraction of SiO${}_{2}$ was 4%, which increased the ORF. However, the ORF decreased when the volume fraction exceeded 4%. Their findings also revealed that the ORF improved as the viscosity and density of the oil decreased, which was achieved by increasing the injected inlet temperature. Specifically, the highest ORF of 37% was obtained when SiO${}_{2}$ was used at a volume fraction of 4% and an inlet temperature of 353.15 K. These findings suggest that using SiO${}_{2}$ NPs at an optimal volume fraction and inlet temperature can significantly improve oil recovery efficiency in a porous medium. This highlights the importance of considering the thermophysical properties of nanoparticles when selecting an optimal nanofluid for enhanced oil recovery applications.

This research suggests a three-dimensional mathematical model that can predict the ORF by simulating the EOR process. In the past, models have focused on how nanoparticles change the wettability of the PM surface and the interfacial tension between the fluids. However, the fluid’s thermophysical properties change due to the nanoparticles, which affects the EOR process, and this aspect has not been previously modeled. The proposed model simultaneously considers the changes in TPs with varying temperatures and VFs using novel empirical correlations for the first time. It is observed that these properties play a critical role in evaluating the efficacy of NF flooding through the oil reservoirs for EOR. These correlations are used to compute the relative permeability formulas for water and oil. Additionally, we investigate how the VF and IT impact the nanofluid TPs and improve EOR performance.

## 2. Mathematical Modeling

Simulating the EOR process requires computational fluid dynamics (CFD) in oil reservoir engineering. This tool allows engineers to model fluid behavior in porous media, leading to process optimization and a better understanding of fluid interactions [39]. CFD examines different methods of EOR such as waterflooding, gas injection, chemical injection, and NF flooding [16]. Engineers can optimize the simulation of injection rates to evaluate their effectiveness. CFD considers factors such as NF properties, fluid compressibility, and fluid–rock interactions, which contribute to the analysis of EOR.

A study utilized COMSOL Multiphysics software version 4 and the finite-element method to simulate NF flooding and assess its impact on the increase in the ORF [40]. Parameters such as pressure, velocity, saturation, and temperature were analyzed in the research [41,42,43]. The equations for continuity, Darcy, energy, and concentration were solved. The empirical correlations determined thermal conductivity, density, and viscosity. Additionally, we examined the effects of NP deposition on porosity and permeability [44].

### 2.1. Governing Equations

To simulate NF flooding in PM for EOR, the mathematical equations usually consist of the two-phase Darcy’s law and equations for energy and concentration. The two-phase Darcy’s law accounts for the fluid’s pressure gradient and viscous forces. The concentration and energy equations account for the concentration of NPs inside water, which refers to the number of NPs present in a given volume of water. The concentration of NPs can vary widely depending on the preparation method, including the size and stability of the NPs and the type of stabilizing agent used. The stability of NPs in water can be influenced by temperature. The equation for energy takes into consideration the transfer of heat that occurs between the fluids and the reservoir’s PM.

The two-phase Darcy’s law is a well-known model that is often used to explain the movement of two fluids that cannot be mixed, such as oil and NF, in a porous material. In particular, when NF flooding is used to enhance OR, the two-phase Darcy’s law can be used to describe how oil and NF flow through the porous rock structure simultaneously. According to this law, there is a direct relationship between the pressure gradient and the velocity of the fluid, and a constant of proportionality known as permeability governs this relationship. The symbol $K_{m}$ represents this constant, indicating how fluids can move through the PM.

The two-phase Darcy’s law considers the relative permeabilities of NFs, $\kappa_{r,nf}$, and water, $\kappa_{r,w}$, which can be affected by the presence of NPs. By incorporating additional models, such as those related to heat and mass transfer, the complete behavior of the NF flooding process can be simulated by combining the two-phase Darcy’s law with these other models. The specific forms of these equations depend on factors such as fluid properties, porosity, permeability, and temperature, among others. The solutions of these equations can provide insights into the flow and transport of NFs in PM, which can be used to optimize EOR processes.

In this section, the two-phase Darcy equations are used to calculate the pressure and velocity distribution of oil and NF. The saturation equation is used to measure the saturation of NF and oil phases and estimate other parameters associated with the NF flooding simulation process. The following equations model the NF flooding process inside the PM.

#### 2.1.1. Transport Equations

Darcy’s law and the continuity equations govern the flow of NF and oil in a PM. The PM has a porosity ε and an absolute permeability (K${}_{m}$). Darcy’s law describes the flow in the PM for each phase, with relative permeabilities $\kappa_{r,i}$ where *i* represents nf and *o*. The subscripts nf and *o* indicate the NF and oil fluids, respectively. The velocities $u_{i}$ are as calculated as follows:

(1) $$u_{nf}=-\frac{K_{m}\kappa_{r,nf}}{\mu_{nf}}\left(\frac{\partial }{\partial x}i+\frac{\partial }{\partial y}j+\frac{\partial }{\partial z}k\right)\left(P_{nf}-\rho_{nf}gz\right),$$

(2) $$u_{o}=-\frac{K_{m}\kappa_{r,o}}{\mu_{o}}\left(\frac{\partial }{\partial x}i+\frac{\partial }{\partial y}j+\frac{\partial }{\partial z}k\right)\left(P_{o}-\rho_{o}gz\right),$$

where $\mu_{nf}$, $\mu_{o}$, $\rho_{nf}$, $\rho_{o}$, $P_{nf}$, and $P_{o}$ are the viscosity, density, and pressure for NF and oil, respectively. The gravity (*g*) can be neglected due to the NF flooding horizontally.

Fluids are assumed to be incompressible. As a result, the two continuity equations and the saturation condition for $S_{nf}$ and $S_{o}$ can be expressed as follows:

(3) $$S_{nf}+S_{o}=1,$$

(4) $$\varepsilon \rho_{nf}\frac{\partial S_{nf}}{\partial t}+\left(\frac{\partial }{\partial x}i+\frac{\partial }{\partial y}j+\frac{\partial }{\partial z}k\right)\cdot \left(\rho_{nf}u_{nf}\right)=0,$$

(5) $$\varepsilon \rho_{o}\frac{\partial S_{o}}{\partial t}+\left(\frac{\partial }{\partial x}i+\frac{\partial }{\partial y}j+\frac{\partial }{\partial z}k\right)\cdot \left(\rho_{o}u_{o}\right)=0.$$

By adding Equation (4) to Equation (5), and then using Equation (3), Equation (6) can be expressed as follows:

(6) $$\left(\frac{\partial }{\partial x}i+\frac{\partial }{\partial y}j+\frac{\partial }{\partial z}k\right)\cdot \left(u_{nf}+u_{o}\right)=0,$$

Then,

(7) $$u_{nf}+u_{o}=u=\mathrm{Constant}\;m/s,$$

where $u_{nf}$ and $u_{o}$ are the NF and oil velocities, respectively.

By adding Equation (1) to Equation (2), and utilizing the fact that $S_{nf}+S_{o}=1$, Equation (7) can be applied, and Equation (8) can be written as

(8) $$u=-K_{m}\left(\frac{\kappa_{r,nf}}{\mu_{nf}}+\frac{\kappa_{r,o}}{\mu_{o}}\right)\left(\frac{\partial }{\partial x}i+\frac{\partial }{\partial y}j+\frac{\partial }{\partial z}k\right)\left(P_{nf}+P_{o}\right).$$

When the NF is injected into the PM, it causes a change in the wettability of the medium from oil-wet to NF-wet. This change requires the inclusion of a capillary pressure correlation that considers the alteration in wettability through empirical relationships.

(9) $$p_{c}=-B_{C}\times \log\left(Se\right),$$

where $B_{C}$ and Se are the effective capillary pressure and saturation parameters, respectively.

(10) $$P_{c}=P_{nf}-P_{o},$$

where $P_{o}$ is a constant, which can be set to 0 without loss of generality. Equation (8) can be reduced to

(11) $$u=-K_{m}\left(\frac{\kappa_{r,nf}}{\mu_{nf}}+\frac{\kappa_{r,o}}{\mu_{o}}\right)\left(\frac{\partial }{\partial x}i+\frac{\partial }{\partial y}j+\frac{\partial }{\partial z}k\right)P_{c}.$$

Equation (11) is solved for $\nabla p_{nf}=\nabla p_{c}$, and the resulting expression is substituted into Equation (1), leading to the derivation of Equation (12):

(12) $$u_{nf}=\left(\frac{\kappa_{r,nf}}{\mu_{nf}}u\right)/\left(\frac{\kappa_{r,nf}}{\mu_{nf}}+\frac{\kappa_{r,o}}{\mu_{o}}\right),$$

By substituting the value of $u_{nf}$ from Equation (12) into Equation (4), Equation (13) can be derived, which is given as follows:

(13) $$\nabla \cdot \left(\rho_{nf}\left(\left(\frac{\kappa_{r,nf}}{\mu_{nf}}u\right)/\left(\frac{\kappa_{r,nf}}{\mu_{nf}}+\frac{\kappa_{r,o}}{\mu_{o}}\right)\right)\right)=-\varepsilon \rho_{nf}\frac{\partial S_{nf}}{\partial t}.$$

In other words, the NF-oil flow (Equation (16)) can be obtained using Darcy’s law (Equations (1) and (2)) and the continuity equations (Equations (4) and (5))

(14) $$\frac{\partial \varepsilon \rho_{nf}S_{nf}}{\partial t}+\nabla \cdot \left(\rho_{nf}S_{nf}u\right)=\nabla \cdot \left(D_{c}\nabla \rho_{nf}S_{nf}\right),$$

(15) $$D_{C}=\frac{\kappa_{r,nf}}{\mu_{nf}}\;K\left(S_{nf}-1\right)\frac{\partial P_{c}}{\partial S_{nf}}.$$

where $D_{c}$ is the capillary diffusion coefficient parameter. As mentioned earlier, the capillary pressure relationship in Equation (10) ($P_{c}$) is utilized in this modeling.

To solve Equation (13), the effective density and viscosity of the NF must be considered since it is a liquid composed of water and NPs. Over the years, several models have been proposed to predict oil production based on empirical correlations of the density and viscosity with various VFs. The proposed model is applicable over a wide range of temperatures and VFs and can be used to determine the density and viscosity of NF as a function of the VF and temperature. As a result, Equations (16) and (17) are utilized to calculate the density and viscosity as a function of the temperature and VF [45]. A novel model based on the density, viscosity, and TC of the NF is proposed to simulate the EOR process:

(16) $$\rho_{nf}=\rho_{w}[1.032+\left(0.03774*\emptyset \right)+\left(-0.0004753*T+\left(-0.00007523*T*\emptyset \right)\right]$$

(17) $$\mu_{nf}=\mu_{w}*\left[1.116+\left(0.3923*\emptyset \right)+\left(\frac{-2.183}{T}\right)+\left(\frac{-3.849*\emptyset }{T}\right)\right]$$

The results obtained using the model are compared to those obtained using the Maxwell [46] and Murshed models [47]. The Maxwell model [46] is used to calculate the TC of the particles.

(18) $$k_{eff}=k_{w}\frac{k_{np}+2k_{w}-2\emptyset \left(k_{w}-k_{np}\right)}{k_{np}+2k_{w}+\emptyset \left(k_{w}-k_{np}\right)}$$

where $k_{np}$, $k_{w}$, and *∅* represent the TC of the NPs, water, and VF, respectively.

The TC of NFs in the Murshed model [47] consists of two parts: static and dynamic

(19) $$k_{eff-nf}=k_{\mathrm{st}}+k_{dy}$$

where

(20) $$\begin{array}{cc} \; & k_{eff-nf}\\ \; & =\left\{k_{w}\frac{\phi_{\mathrm{np}}\omega \left(k_{\mathrm{np}}-\omega k_{w}\right)\left[2\gamma_{1}^{3}-\gamma^{3}+1\right]+\left(k_{\mathrm{np}}+2\omega k_{w}\right)\gamma_{1}^{3}\left[\phi_{\mathrm{np}}\gamma^{3}\left(\omega -1\right)+1\right]}{\gamma_{1}^{3}\left(k_{\mathrm{np}}+2\omega k_{w}\right)-\left(k_{\mathrm{np}}-\omega k_{w}\right)\phi_{\mathrm{np}}\left[\gamma_{1}^{3}+\gamma^{3}-1\right]}\right\}\\ \; & +\left\{\phi_{\mathrm{np}}^{2}\gamma^{6}k_{w}\left(3\Lambda^{2}+\frac{9\Lambda^{3}}{16}\frac{k_{\mathrm{cp}}+2k_{w}}{2k_{\mathrm{cp}}+3k_{w}}+\frac{3\Lambda^{4}}{2^{6}}\right)\right\}\\ \; & +\left\{\frac{1}{2}\rho_{\mathrm{cp}}c_{\mathrm{np}-\mathrm{cp}}d_{s}\left[\sqrt{\frac{3K_{B}T\left(1-1.5\gamma^{3}\phi_{p}\right)}{2\pi \rho_{\mathrm{cp}}\gamma^{3}r_{\mathrm{np}}^{3}}}+\frac{G_{T}}{6\pi \eta \gamma r_{\mathrm{np}}d_{s}}\right]\right\}\\ \; \end{array}$$

where $\gamma =1+\frac{t}{r_{p}}$ and $\gamma_{1}=1+\frac{t}{2r_{p}}$, and a complex particle TC is defined by:

(21) $$k_{\mathrm{cp}}=k_{\mathrm{lr}}\frac{2\left(k_{\mathrm{np}}-k_{\mathrm{lr}}\right)+\gamma^{3}\left(k_{\mathrm{np}}+2k_{\mathrm{lr}}\right)}{\left(k_{\mathrm{lr}}-k_{\mathrm{np}}\right)+\gamma^{3}\left(k_{\mathrm{np}}+2k_{\mathrm{lr}}\right)}$$

The TC of the nanolayer can be expressed as $k_{\mathrm{lr}}=\omega k_{f}$, where ω is an empirical parameter that is influenced by the order and shape of the molecules present in the interface of the NPs, as well as their nature and surface chemistry (where ω>1). The value of Λ is defined as:

(22) $$\Lambda =\frac{k_{\mathrm{cp}}-k_{f}}{k_{\mathrm{cp}}+2k_{f}}$$

where $G_{T}$ represents the total energy that exists between two NPs, which is mathematically defined as:

(23) $$\begin{array}{cc} G_{T}= & -\frac{A}{6}\left[\frac{2r_{\mathrm{cp}}^{2}}{d_{s}^{2}+4r_{\mathrm{cp}}d_{s}}+\frac{2r_{\mathrm{cp}}^{2}}{d_{s}^{2}+4r_{\mathrm{cp}}d_{s}+4r_{\mathrm{cp}}^{2}}\right.\\ \; & \left.+\ln\left(\frac{d_{s}^{2}+4r_{\mathrm{cp}}d_{s}}{d_{s}^{2}+4r_{\mathrm{cp}}d_{s}+4r_{\mathrm{cp}}^{2}}\right)\right]+2\pi \varepsilon_{r}\varepsilon_{0}r_{\mathrm{cp}}\zeta^{2}\exp\left(-\kappa d_{s}\right) \end{array}$$

The Murshed model is defined in detail in [47].

The RP incorporates the necessary saturation ($S_{e}$) for determining the ORF, which can be calculated as

(24) $$S_{e}=\frac{S_{nf}-S_{rnf}}{1-S_{rnf}-S_{ro}},$$

where ($S_{rnf}$) and ($S_{ro}$) represent the residual saturation of the NF and oil, respectively.

#### 2.1.2. RPs of NF and Oil

When NPs are retained, they cause the wettability of the PM to shift from oil-wet to NF-wet, which leads to an expansion of the RP correlations to accommodate the mixed-wet conditions. Equations (25) and (26) are used to represent the RPs of both the NF and oil [48].

(25) $$k_{rnf}=3S_{e}+\frac{2}{\lambda },$$

(26) $$k_{ro}=\left(1-S_{e}^{2}\right)\times \left(1-S_{e}^{1+\frac{2}{\lambda }}\right),$$

where λ represents the index used to describe the size of the pores.

#### 2.1.3. Nanoparticle Transport in PM

The transport of NPs within the PM throughout each phase of the flow is determined using the following equation [49]:

(27) $$u\frac{\partial C_{i}}{\partial t}+\varepsilon S_{w}\frac{\partial C_{i}}{\partial t}-\frac{\partial }{\partial x}\left(\varepsilon S_{w}D_{c}\frac{\partial C_{i}}{\partial x}\right)+R_{i}=0,$$

(28) $$D_{c}=\frac{k_{rw}}{\mu_{w}}k\left(S_{w}-1\right)\frac{\partial P_{c}}{\partial S_{w}},$$

where the concentration, diffusion, and NP rate loss in the PM are denoted by $C_{i}$, $D_{c}$, and $R_{i}$, respectively.

The term $R_{i}$ can be divided into two terms:

- The number of NPs on the surface.

- The number of NPs located in the throat of the PM.

(29) $$R_{i}=\frac{\partial \sigma }{\partial t}+\frac{\partial \sigma^{*}}{\partial t},$$

where $\sigma^{*}$ and σ represent the numbers of NPs. Specifically, $\sigma^{*}$ refers to the number of NPs confined to the PM’s throat (presumably some particle filter), and σ refers to the number of NPs deposited on the PM’s surface per unit volume.

(30) $$\frac{\partial \sigma }{\partial t}=k_{d}uC,$$

(31) $$\frac{\partial \sigma^{*}}{\partial t}=k_{p}uC,$$

where $k_{d}$ and $k_{p}$ represent constants related to the deposition and entrainment of NPs, respectively. The vector u represents the required phase velocity of the NPs, which is 0.0025 m/s.

When NPs are deposited on the surface of a pore, it can cause the pore’s opening to become obstructed, resulting in a decrease in the PM’s overall ability to allow fluids to pass through it, as well as a reduction in the amount of space within the PM. The equations given below can be used to determine these values:

(32) $$\varepsilon =\varepsilon_{0}-\left(\sigma +\sigma^{*}\right),$$

where

(33) $$K=K_{0}{\left[\left(1-F\right)\alpha_{f}+\frac{F\varepsilon }{\varepsilon_{0}}\right]}^{n},$$

In the given context, $\varepsilon_{0}$, $K_{0}$, *K*, ε, *n*, $\alpha_{f}$, *F*, $\alpha_{f}$, *n*, and *F* are used to represent specific physical properties or parameters. $\varepsilon_{0}$ denotes the permeability, $K_{0}$ represents the porosity, *K* represents the fluid seepage constant, and ε represents the percentage of the original cross-sectional area that is available for fluid flow. The value of ε can range from 0 to 1.

The variable *n* is an empirical factor that can be any value between 1 and 3. Additionally, $\alpha_{f}$ and *F* denote the percentage of the initial cross-sectional area available for the flow of the NF. Although the value of $\alpha_{f}$ is constant, the value of *F* can vary.

The TPs of the oil and water are shown in Table 1. The properties of the NPs (SiO${}_{2}$) and the PM are provided in Table 2, which are required for the computation of the model.

#### 2.1.4. Heat-Transfer Equation

The equations for heat transfer in particulate materials (PM) are employed to investigate the heat transfer state in the PM. These equations utilize the effective TPs of the PM, consisting of the nanofluid and oil phases.

(34) $${\left(\rho_{tot}C_{p}\right)}_{\mathrm{eff}\;}\frac{\partial T}{\partial t}+\rho_{tot}C_{p}u\cdot \nabla T+\nabla .q=Q$$

(35) $$q=-k_{\mathrm{eff}\;}\nabla T$$

(36) $${\left(\rho C_{p}\right)}_{\mathrm{eff}\;}=\theta_{p}\rho_{p}C_{p,p}+\left(1-\theta_{p}\right)\rho C_{p}$$

(37) $$k_{\mathrm{eff}\;}=\theta_{p}k_{s}+\left(1-\theta_{p}\right)k_{tot}$$

where $\theta_{p}$ is the VF of the porous rock.

#### 2.1.5. Boundary and Initial Conditions

The velocity and inlet temperature of the NF injected into the PM through the inlet side are defined as follows:

Inlet Velocity Condition:

(38) $$n\cdot \rho_{o}u\mathrm{nf}0=0.01\;m/s,$$

where n represents the unit normal vector, $\rho_{o}$ is the density of the NF, and unf0 denotes the initial velocity of the NF.

Inlet Temperature Condition:

(39) $$T=T_{\mathrm{in}}$$

where *T* is the temperature and $T_{\mathrm{in}}$ is the specified inlet temperature.

At the beginning of the flooding process (t=0) inside the PM, the NF saturation and the pressure at the outlet are defined as follows:

Outlet Pressure Condition:

(40) $$p=p_{\mathrm{nf}}=0,\mathrm{Pa}$$

where *p* represents the pressure and $p_{\mathrm{nf}}$ denotes the NF pressure at the outlet.

NF Saturation Condition:

(41) $$S_{\mathrm{nf}0}=0\;\mathrm{at}\;t=0$$

where $S_{\mathrm{nf}0}$ represents the initial NF saturation.

The conditions at the cylinder outlet side are defined as follows:

Outlet Velocity Condition:

(42) $$-n\cdot \rho_{o}uo=\rho_{o}uo0$$

where uo represents the velocity at the outlet, and uo0 denotes the specified initial outlet velocity.

Temperature Gradient Condition:

(43) $$-n\cdot k_{\mathrm{eff}}\nabla T=0$$

where $k_{\mathrm{eff}}$ is the effective thermal conductivity, and ∇T represents the temperature gradient.

The initial conditions for the simulation are defined as follows:

Initial NF Saturation:

(44) $$S_{\mathrm{nf}}=0$$

Initial Wetting Phase Saturation:

(45) $$S_{o}=1$$

Initial Pressure:

(46) $$P_{o}=0$$

Initial Temperature:

(47) $$T_{\mathrm{init}}=293.15,K$$

Surface Tension:

(48) $$\sigma =\sigma^{*}=0$$

In addition, the initial concentration of the nanoparticles (NPs) in the PM is assumed to be zero.

Initial Concentration of the NPs:

(49) $$C_{\mathrm{init}}=0\;\mathrm{at}\;t=0$$

These conditions comprehensively describe the initial and boundary conditions for the problem under consideration.

### 2.2. Geometry

**Figure 1:**
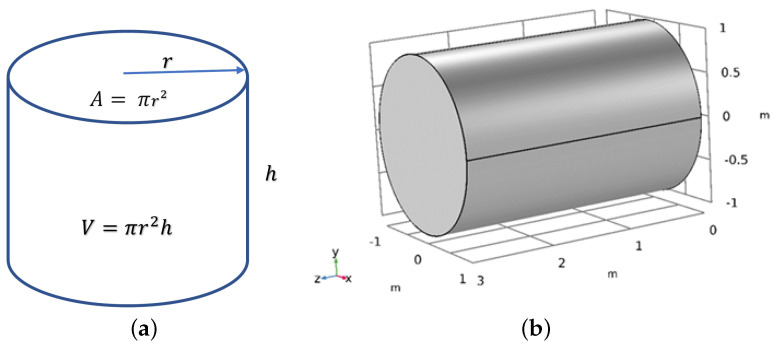
(**a**) Cylinder properties, and (**b**) model geometry.

**Figure 2:**
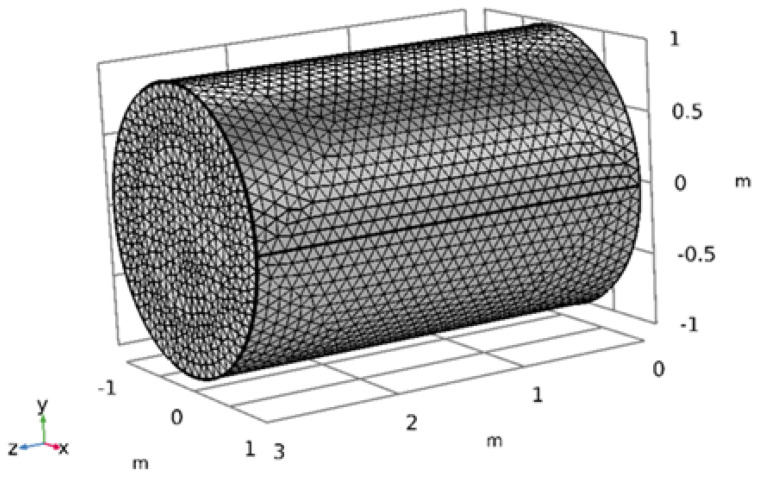
Mesh geometry.

This research involves the simulation of the impact of an NF on the ORF. Previous studies have utilized different NPs mixed with water as NFs to enhance the ORF by injecting them into the PM to force out the oil flow, thereby increasing oil production. Table 3 presents the properties of the PM used in this study, which is a cylinder-shaped oil reservoir modeled using COMSOL Multiphysics software and based on the properties of an actual reservoir. The geometry and properties used in the current study are depicted in Figure 1a and Figure 1b, respectively, and the boundary conditions for the problem are shown in Table 4. The NF layer closest to the surface remains stationary as it moves through the PM, and the triangular mesh in the CMS software version 6 is suitable for meshing the NF flow in the PM, as shown in Figure 2.

### 2.3. Grid Independence

It was essential to consider the following steps in selecting the optimal mesh for EOR modeling to achieve accurate and reliable results. The first step was to determine the spatial resolution requirements. This involved evaluating the physical and chemical processes involved and determining the minimum size of the elements required to capture the relevant features of the system. This is important because the accuracy of the simulation results depends on the mesh’s spatial resolution.

Next, a balance needed to be struck between accuracy and computational efficiency. The mesh needed to have elements of sufficient size to provide accurate results. However, it was also essential to consider the computational resources available and the cost-effectiveness of the simulation. The goal was to select a mesh that met the accuracy requirements while being computationally efficient.

It was also essential to consider the grid’s adaptability. The mesh needed to be able to adapt to changes in the reservoir’s physical properties over time such as variations in fluid saturation and pressure. This ensured that the simulation results remained accurate and relevant over time. Then, the mesh was validated, which involved comparing the simulation results with actual field data and refining the mesh to improve accuracy. By validating the mesh, it is possible to ensure that the simulation results are accurate and reliable.

Finally, six meshes with different grid elements (30,000, 40,000, 50,000, 60,000, and 70,000) were developed to examine grid independence. The model was run six times using water, and the parameters and properties of the water and oil, which are shown in Table 1, were fixed to compare the six ORF values and determine the optimal grid.

**Figure 3:**
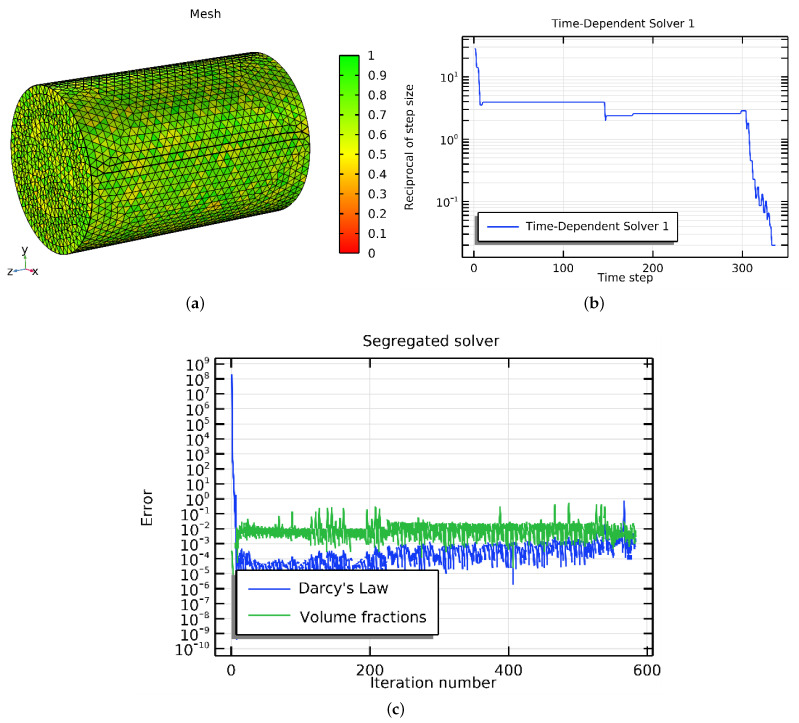
(**a**) Inspection of the mesh, (**b**) convergence plot, and (**c**) error of less than 1 × $10^{-5}$.

During the simulation, the NF was introduced into the reservoir through the inlet at a velocity of 0.01 m/s, and the oil pressure at the outlet was maintained at zero. The reservoir was configured so that the oil and NF saturations were 1 and 0, respectively. In addition, the remaining amount of oil left in the reservoir, known as the residual oil saturation, was only 0.2%. Table 5 presents the mesh with varying numbers of elements and the ORF value at a specific point (2 m height, 0.5 m radius, 600 s). In other words, the percentage of oil recovered from a particular area was calculated using a measurement point located 1 m away from the area. Measurements were taken at intervals of 0.5 m in two different directions, and the data were collected for 200 s. Various grid sizes were used for obtaining the measurements. The results showed that the ORF values remained primarily unchanged after mesh number 4, as increasing the number of elements led to longer computation times. Therefore, mesh number 4 was selected as the best option and used for all simulations in the study. In addition, the CMS generated a geometry mesh plot demonstrating the quality of the mesh elements. The mesh elements were considered of good quality if their color was green with a value of 1, indicating the highest quality. Figure 3a reveals that the meshing quality of the current study’s geometry is good, as all elements are green. Furthermore, Figure 3b depicts the convergence of the governing equations at a time interval of 400 s. The error was less than $10^{-5}$, which is depicted in Figure 3c.

#### Assumptions

- The NF (NP-water) is homogeneous, meaning it is considered one phase. The thermophysical parameters of the NF are computed using the experimental models.
- The PM is homogeneous, contributing to lower simulation processing costs.
- The NF and PM have incompressible flows.
- The EOR process is modeled using the two-phase Darcy’s law.
- The flow and PM are considered to be isothermal.

### 2.4. Verification

**Figure 4:**
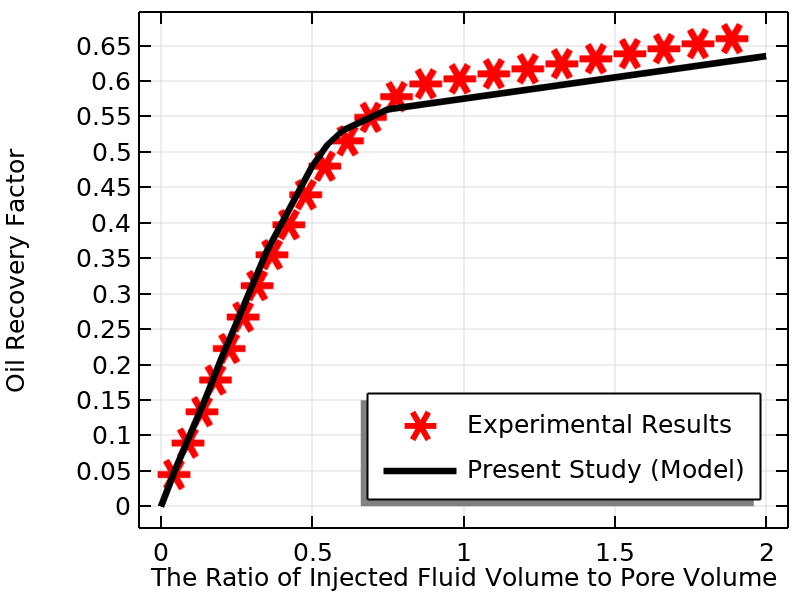
An examination of how data from the numerical analysis compared to data collected through practical experiments.

In this study, we developed a model to simulate NF flooding in a three-dimensional PM. To validate our model, we compared our numerical findings with the laboratory results obtained by Minakov et al. [54], who conducted an experiment using NF (water-SiO${}_{2}$). The characteristics of the PM are listed in Table 6. Our validation results, depicted in Figure 4, demonstrated a close agreement between our simulation and experimental data, with a relative error of only 2%.

## 3. Results and Discussion

Different mesh elements were used to solve the model geometry, and the variation in the ORF was compared. Mesh number 4, which had ideal elements, was selected for all modeling stages. After achieving good agreement between the numerical and experimental data, we analyzed the effects of various parameters. Specifically, we examined how the VF range of 1–5% at different inlet temperatures (293.15–500 K) affected the nanofluid’s thermophysical properties for EOR.

### 3.1. The Effects of Nanofluid’s TPs on EOR

During NF flooding at varying ITs, we analyzed how the addition of five different VFs to water affected the nanofluid’s TPs. The VF of the NPs is a significant factor that can impact the TC of the NF. It is a common understanding that a higher VF results in an increased TC due to NPs acting as conductors. As the concentration of NPs in the fluid increases, the overall conductivity of the fluid improves.

The impact of the inlet temperature on the TC of the NF is a multifaceted issue that depends on various factors, including the type of NPs and base fluid, the size and form of the NPs, and the temperature range. Remarkably, the NF’s thermal conductivity increases as the IT increases. Nonetheless, in some scenarios, the TC of the NF may decrease as the temperature rises, a phenomenon referred to as TC depression. The NPs then aggregate at higher temperatures, reducing their ability to effectively conduct heat.

**Figure 5:**
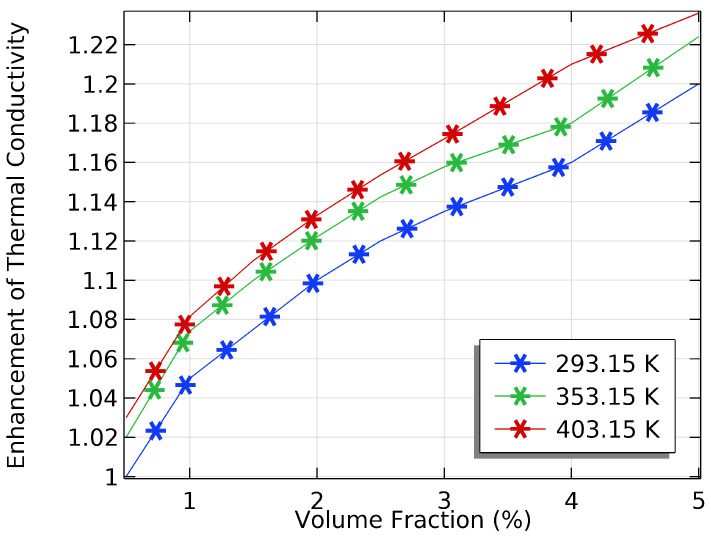
The effect of VF at different inlet temperatures on the nanofluid TC.

**Figure 6:**
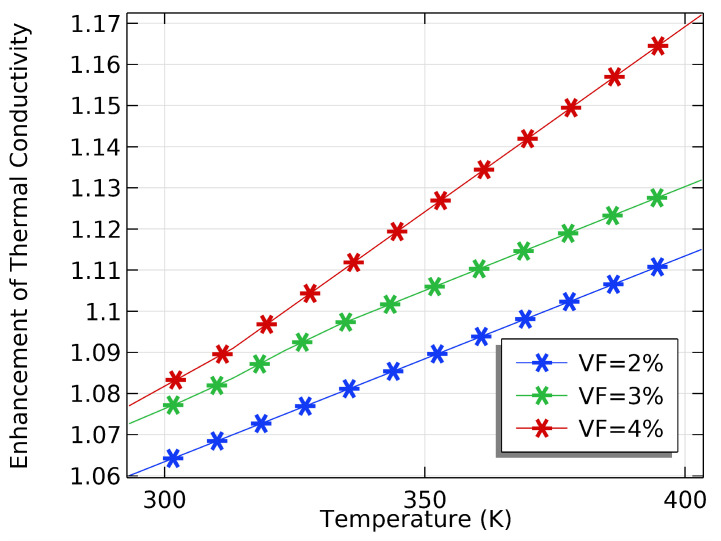
The effect of inlet temperature at different VFs on the nanofluid’s TC.

We investigated how VFs and inlet temperatures can improve thermal conductivity and enhance oil recovery in the petroleum industry. Various factors, including the VF and IT, influenced the conductivity of the NF. Figure 5 shows that the increase in the VF and IT significantly increased the thermal conductivity. Furthermore, Figure 5 and Figure 6 show that the addition of VFs improved the thermal conductivity compared to pure water. As the VF and IT increased, the thermal conductivity of the NF increased, leading to an increase in oil recovery. However, the gradual increment in thermal conductivity can be attributed to the tendency of NPs to agglomerate, which reduced the effectiveness of the NF on EOR. The IT also affected the conductivity of the NF. At higher ITs, the thermal conductivity of the NF increased due to increased Brownian motion, which helped break up agglomerates and improve particle dispersion. Therefore, the effect of VF and IT on NFs is crucial to enhancing oil recovery. However, very high temperatures and VFs caused a decrease in thermal conductivity, resulting in a decrease in oil recovery. Nanofluids are helpful in the EOR process as they have tailored thermal properties. One advantage of nanofluids is that they can improve oil extraction efficiency by increasing the thermal conductivity and heat transfer rate of the fluid that heats the reservoir. Adding SiO${}_{2}$ to the base fluid (water) can significantly improve the TC, as SiO${}_{2}$ has a high TC. The enhanced heat transfer rate reduces the energy required to heat the reservoir, resulting in cost savings and improved OR rates. When nanoparticles are added to water, they can change the density and thickness of the fluid. This can improve sweeping efficiency, increase oil recovery, and reduce the production of non-formation fluids. To determine the effectiveness of using NPs for enhanced oil recovery, it is essential to measure the VF, which is the proportion of NPs in an NF to the entire volume of the NF. A higher VF usually means better performance for EOR but it can also lead to increased viscosity, which can cause issues with injection and flow. The NF’s inlet temperature is crucial to its performance in EOR. The NF’s density, viscosity, and thermal conductivity affect its ability to transfer heat, which impacts the inlet temperature. By increasing the IT, the viscosity of the NF can be reduced, improving its flow properties and making it more effective for sweeping the reservoir.

**Figure 7:**
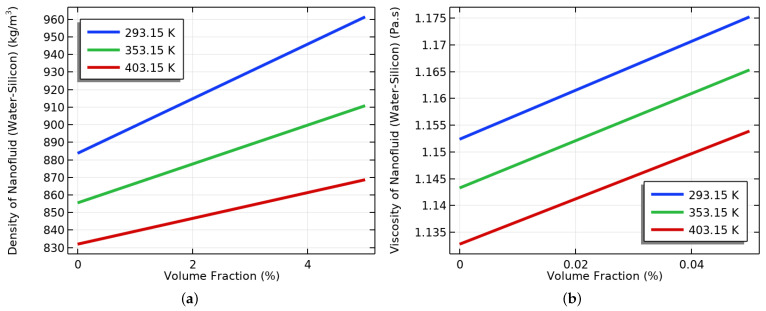
(**a**) The effect of VF at different inlet temperatures on density, and (**b**) viscosity.

**Figure 8:**
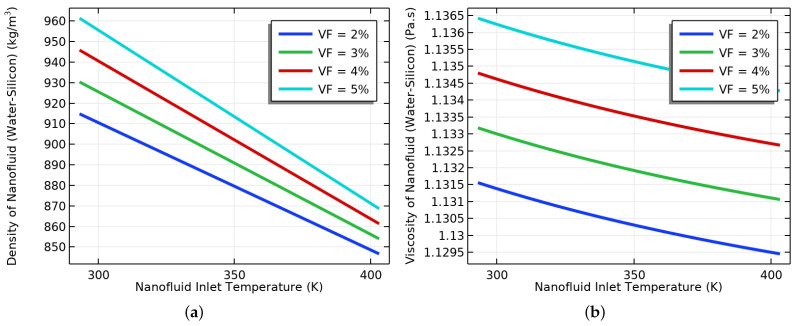
(**a**) The effect of the inlet temperature at different VFs on density, and (**b**) viscosity.

To optimize the performance of NFs in EOR, it is crucial to simultaneously consider their VF and IT. Our research delved into the impact of these factors on the NF’s performance and identified an optimal VF and IT that can boost oil production. Increasing the VF of the base fluid resulted in an increase in the NF’s density and viscosity, as shown in Figure 7a,b. Conversely, increasing the IT reduced the density and viscosity of the NF, as demonstrated in the same figures. However, Figure 8a,b illustrate that an increase in the VF led to an upsurge in the density and viscosity. Our study found that a VF of 0.4% and an IT of 353.15 K were the most effective in enhancing the TC of the NF and improving its performance in EOR.

We studied how the IT and VF affect the density and viscosity of the fluid used in EOR. We looked at different factors such as the properties of the oil and the reservoir. Adding NPs increased the viscosity and density of the fluid since it improved the interaction between the fluid and the rock reservoir. However, the effects of the VF on the density and viscosity varied depending on the size, shape, and surface charge of the NPs used. We studied how temperatures and VFs can affect the density and viscosity of the NF used in EOR. Our results showed that when the VF increased, the fluid’s viscosity also increased, which was more noticeable at higher temperatures. As a result, although higher temperatures led to lower densities, adding VFs increased the viscosity. There have been advancements in the field that involved improving the IT of the fluid used for EOR, leading to a decrease in the fluid’s density due to thermal expansion and an increase in viscosity due to reduced intermolecular forces at higher temperatures. As a result, the improved IT led to increased fluid mobility and a remarkable ability to recover oil from the reservoir. Furthermore, adding NPs to the EOR fluid affected the density and viscosity of the water, ultimately increasing the fluid’s viscosity and allowing for more efficient oil displacement from the reservoir.

The effects of the VF and IT on the density and viscosity of the NF were examined. As shown in Figure 7a, we discovered that the density of the NF increased with the increase in the VF, irrespective of the IT value. The lowest density was observed at 403.15 K, whereas the highest was observed at 293.15 K. Additionally, Figure 7b shows that viscosity increased with the increase in the VF. The highest viscosity was observed at an IT of 293.15 K, whereas the lowest was observed at an IT of 403.15 K. Therefore, we can conclude that both the VF and IT impact the TPs of nanofluids.

The effect of the IT on the density and viscosity of the NF was analyzed. We utilized water-containing NPs with different volume fractions and determined the density of the NF using the empirical correlation provided in Equation (16). Figure 8a displays that as the IT increased, the density of the NF also decreased. We found that the NF density was lowest at a VF of 2% compared to other VFs, whereas the highest density was observed at a VF of 5%. Moreover, we investigated the impact of the IT on the viscosity of the NF with varying VFs. To calculate the viscosity, we used Equation (17). Figure 8b depicts that the NF viscosity decreased as the IT increased. A VF of 2% had the lowest viscosity compared to the other VFs, whereas a VF of 5% had the highest viscosity compared to the other VFs. We used empirical correlations of the TC, density, and viscosity to study the changes in the properties of the oil and NF. We also examined the continuity equations of both fluids. By adding SiO${}_{2}$ to the NF, we were able to increase the viscosity of the fluid. This improved the fluid flow in the reservoir and helped enhance the OR rates.

### 3.2. How Pressure and Velocity Are Distributed within the PM While Flooding with Nanofluid

To increase oil recovery, NF flooding injection into the oil reservoir for 10 min replaces the original oil. Various factors, including the properties of the base fluid and NPs, the size and shape of the pores in the PM, and the speed of NF injection, affect the pressure and velocity of the NF mixture. The model proposed in this study calculates the pressure using the continuity equation and the velocity using the Darcy equation while considering the initial and boundary conditions.

In enhanced oil recovery, the movement of nanofluid through porous media is smooth and steady, which is known as laminar flow. This occurs because the nanofluid has a low Reynolds number, meaning that the flow is mainly based on viscosity rather than inertia. As a result, the flow pattern is stable and organized due to the relatively weak inertial forces compared to the viscous forces.

**Figure 9:**
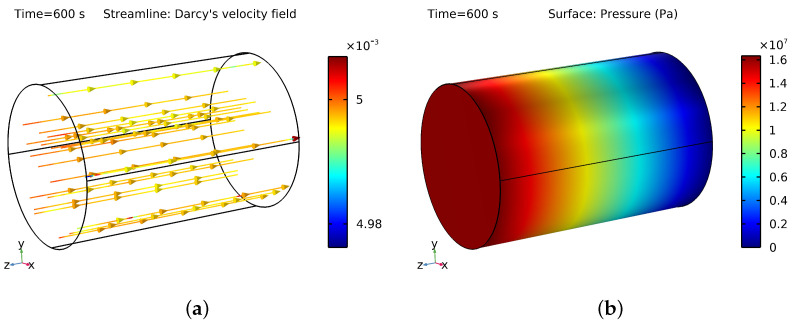
(**a**) Pressure at 600 s. (**b**) The nanofluid’s Darcy velocity at 600 s inside the porous medium.

The visual representation in Figure 9a shows the distribution of the nanofluid velocity in the porous medium during the oil phase, demonstrating how the nanofluid flowed evenly and continuously through the interconnected pores of the medium, leading to the effective displacement of the oil. An important factor to consider is the stability of nanofluid pressure over time. Because of its slow movement through the porous medium, there were minimal pressure fluctuations. This can be seen in Figure 9b, which shows a consistent pressure profile over time. The controlled movement of the nanofluid helped in maintaining a relatively constant pressure, which is crucial for an efficient and effective EOR process.

### 3.3. NF and Water Saturation in PM

Water saturation refers to water in a porous material’s empty spaces or pores. When water flows through the PM, it fills some of the empty spaces while the remaining spaces are filled with oil. The behavior of a nanofluid in the PM is influenced by the properties of the nanoparticles, volume fraction, and characteristics of the PM. Adding NPs to water affects its properties, including increasing its thermal conductivity and reducing its viscosity, which can lead to changes in the fluid flow behavior in the PM.

**Figure 10:**
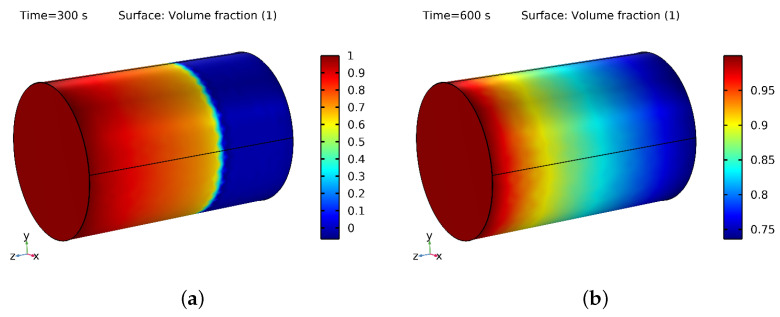
(**a**) Saturation of water at 300 s and (**b**) 600 s.

**Figure 11:**
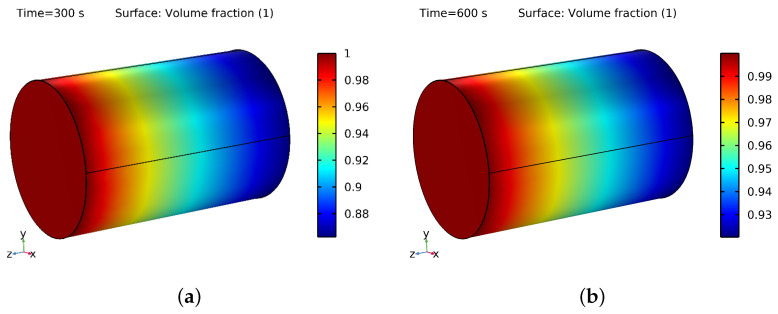
(**a**) Saturation of NF at 300 s and (**b**) 600 s.

Figure 10a,b show the 3D layout of the water and oil phases during the 300 s and 600 s flooding processes. The color red represents water, whereas the color blue represents oil saturation. Figure 10b shows that the displacement of oil by water was more significant at 600 s than at 300 s, as seen in Figure 10a. Additionally, in Figure 10a,b and Figure 11a,b, it can be seen that the saturation of the NF was higher at 300 s and 600 s compared to the saturation of water at the same time intervals. The NF saturation in the PM was higher because of the NPs present in the base fluid, which helped the fluid fill the pores more effectively. This resulted in more NF being retained in the PM, leading to a higher saturation. Additionally, the NPs occupied a more significant portion of the pore space on the PM’s surface, increasing adsorption and further boosting saturation. However, it is essential to note that the saturation level depends on various factors such as the type of PM, the VF of the NPs, the IT, and the fluid itself [55,56].

The saturation of water containing SiO${}_{2}$ with a VF of 4% was more significant, which occupied some of the void spaces and reduced the practical pore volume available for water. Additionally, the surface properties of the SiO${}_{2}$ NPs affected their interaction with the pore surfaces of the PM, further decreasing the effective pore volume available for water. This contributed to a higher water saturation in the SiO${}_{2}$ than in the PM.

The saturation level of the NF comprising SiO${}_{2}$ with a VF of 4% and an inlet temperature of 353.15 K was higher than the water saturation in the PM for various reasons. Firstly, NFs have different TPs than water, meaning that the addition of NPs to water can increase its TC, thereby enhancing its heat transfer performance. As a result, the NF cools down more rapidly than pure water, resulting in a higher saturation level. Secondly, the nanoparticles in the nanofluid can alter the surface properties of the PM. The NPs adsorb onto the pore surfaces of the medium, changing their wettability and resulting in changes in the fluid flow and saturation level. In some cases, the NPs can also coat the PM’s surface, reducing the effective pore size and increasing capillary forces, which leads to a higher saturation level. Thirdly, the saturation level of the nanofluid can also be influenced by its temperature. The thermal expansion of the NF at higher elevated temperatures increases its volume, leading to a higher saturation level.

By combining these factors, the saturation level in the NF containing silicon with a VF of 4% and an inlet temperature of 353.15 K was higher than the water saturation in the PM. Notably, the saturation level depends on the nanofluid’s specific properties, the characteristics of the porous medium, and the conditions of their interaction.

### 3.4. The Effect of VF at Different Inlet Fluid Temperatures on ORF

**Figure 12:**
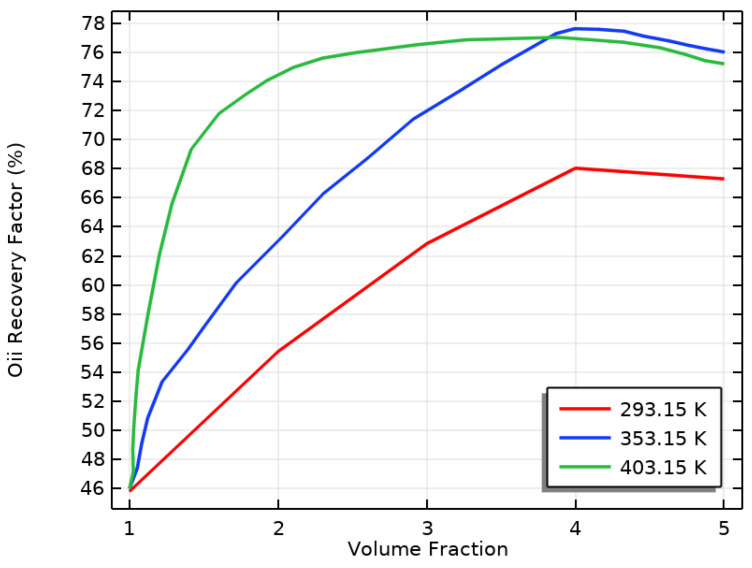
The effect of VF on the ORF at different NF inlet temperatures.

The number of NPs in water can impact the effectiveness of EOR methods. When SiO${}_{2}$ was added, the fluid’s viscosity increased, which helped move oil through the PM, resulting in a higher recovery factor. Increasing the volume fraction made the fluid more viscous, which improved the ORF, as shown in Figure 12. However, when the volume fraction was too high, the fluid became too thick to be effectively injected into the rock, thereby decreasing the ORF. Figure 12 shows that the ORF decreased when the volume fraction exceeded 4%.

As seen in Figure 12, using an NF in EOR resulted in increased ORF when the fluid temperature at the inlet was raised from 293.15 K to 353.15 K. Several factors contributed to this improvement, including the increased fluid viscosity, reduced oil mobility ratio, and changed wettability of the reservoir rock. One mechanism that played a significant role was thermo-viscous displacement, which was influenced by the temperature. As the IT of the nanofluid increased, its viscosity increased, creating more resistance to oil flow and leading to more efficient oil displacement from the reservoir rock. This led to a higher ORF. Moreover, the higher temperature reduced the oil’s viscosity, making it more mobile and thermally activated in the petroleum matrix.

It is important to consider how the IT affects the effectiveness of NPs in oil recovery. Figure 12 shows that higher ITs can increase OR rates as viscosity decreases and oil mobility increases. However, the optimal temperature may vary depending on the specific properties of the oil and NPs. To fully utilize NPs for OR to their fullest potential, it is crucial to carefully examine the impact of the VF on the ORF, which encompasses both the VF and its influence. When the temperature was at 293.15 K, the oil recovery factor increased as the silicon oxide content increased from 1% to 5%, as seen in Figure 12. This is because lower temperatures tend to increase viscosity and resistance to flow. Adding silicon oxide decreased the interfacial tension between the oil and the rock, allowing for more effortless flow and improving the recovery rate. At 353.15 K (80 °C), the higher temperature reduced the oil’s viscosity. Adding silicon oxide also positively reduced the interfacial tension and enhanced OR efficiency, resulting in a higher recovery rate, as shown in Figure 12.

### 3.5. The Effect of NF Inlet Temperature on the ORF at Different VFs

**Figure 13:**
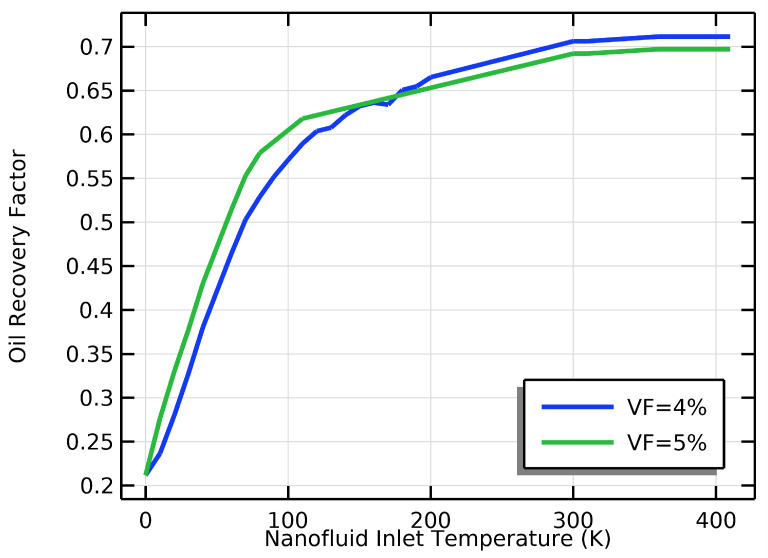
The effect of the inlet temperature of the NF on the ORF at VFs of 4% and 5%.

Increasing the inlet temperature of the NF boosted its thermal conductivity, resulting in better heat transfer performance, as shown in Figure 6. Increasing the inlet temperature for water-silicon oxide NPs with a volume fraction of 4% or 5% likely positively affected their thermal conductivity. At a VF of 4% and a temperature of 353.15 K, the OR increased by 30%, as indicated in Figure 13, before decreasing.

When nanoparticles are well dispersed and do not form agglomerates, increasing their volume fraction in a nanofluid can increase thermal conductivity. However, when the VF of silicon oxide nanoparticles was constant and the inlet temperature of the NF was increased from 0 to 403.15 K, the effect on thermal conductivity was less noticeable than when the VF was increased to 4%. In addition, increasing the IT from 0 to 403.15 K at a VF of 4% produced more oil recovery compared to a VF of 5%.

The temperature of a specific fluid had a notable impact on the ability to extract oil from the reservoir. As the temperature increased, the oil became less viscous, improving its ability to move outside the reservoir, as demonstrated in Figure 13. However, if the temperature became too high, the nanofluid became less viscous, decreasing its ability to push the oil outside and flow through the reservoir. Therefore, the ideal temperature range for this particular fluid is between 293.15 and 353.15 K. This range resulted in the highest oil recovery factor, as shown in Figure 13. These findings suggest that using this fluid in enhanced oil recovery methods can significantly improve the oil recovery factor compared to traditional fluids. Generally, the recovery factor increases as the fluid’s temperature increases, but the oil recovery factor starts to decline once the volume fraction reaches 5% at temperatures higher than 353.15 K.

The effect of the inlet temperature of the nanofluid on the oil recovery factor depends on the nanofluid volume fraction used. When the VF was 4%, increasing the IT of the NF caused a slight increase in the ORF compared to a VF of 5%, as shown in Figure 13. The higher temperature reduced the oil’s viscosity, making it easier to extract from the reservoir. Moreover, when the VF of the NF was 4%, the effect of the IT on the ORF was more pronounced. This is because the higher VF of the NF improved the heat transfer and enhanced convection, reducing the oil’s viscosity and improving its recovery. Therefore, it is essential to consider these factors to accurately predict the effect of the IT of the NF on the ORF.

### 3.6. Comparison of ORF of Water and NF

**Figure 14:**
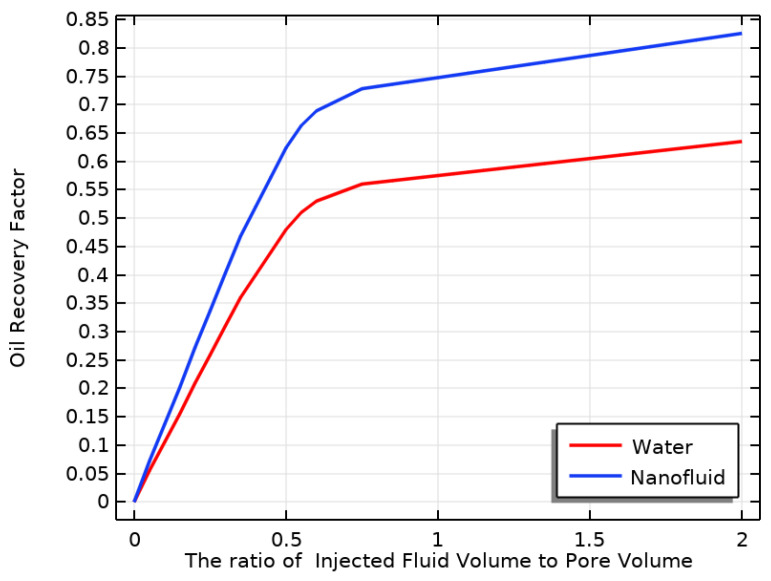
Comparison of ORF of water and nanofluid.

The simulation was performed twice, comparing the effects of water-SiO${}_{2}$ and water on increasing the ORF. The results in Figure 14 indicate that the use of the NF (water-SiO${}_{2}$) led to a more significant increase in the ORF than water flooding. The mixture of water-SiO${}_{2}$ produced the highest ORF, which was 30% higher than that of water flooding. In the case of water-SiO${}_{2}$ flooding, NPs increased OR in the PM compared to pure water flooding for several reasons.

The addition of NPs to water resulted in the increased viscosity of the NF, as demonstrated in Figure 7b and Figure 8b. This led to better sweep efficiency and decreased mobility of the injected fluid, ultimately facilitating the movement of oil through the PM and toward the production well. Additionally, NPs improved the TC of the injected water, as evidenced in Figure 5 and Figure 6. This helped reduce the oil’s viscosity and improve its mobility within the reservoir.

When NPs absorb rock and modify it, they alter its surface properties. By using a water-SiO${}_{2}$ NF, the NPs enhanced the rock surface’s ability to be wetted by water, allowing for the easier displacement of oil from the pores by water. The NPs also decreased the interfacial tension between the oil and the water, causing oil droplets to detach more easily from the rock surface and move toward the production well. When using a water-SiO${}_{2}$ NF with a VF of 4% at 353.15 K, OR in the PM was higher compared to pure water flooding due to these mechanisms. However, the specific effects of NF flooding can vary depending on factors such as the reservoir rock type, injection rate and pressure, and properties of the NF.

## 4. Conclusions

In summary, the injection of an NF into a 3D porous medium for EOR has been modeled. The FEM has been used to compute the governing equations, Darcy’s law, mass conservation, concentration, and energy equations, coded in COMSOL software. The inlet temperature, VF, and pressure were shown to influence the nanofluid’s TPs in the PM. Accurately considering these TP changes in modeling the EOR process is crucial for predicting the ORF. This study presented a mathematical model that can describe the changes in the density, viscosity, and TC of nanofluids with variations in the temperature and VF. This information is crucial for developing efficient and cost-effective EOR methods using NFs. The findings of this study provide a foundation for further research in this field and can contribute to the advancement of technology in the oil and gas industry.

The results can be summarized as follows:

- Nanofluid flooding in a 3D porous medium for the EOR process has been modeled.
- The proposed model addresses the modifications in thermophysical properties concurrently with fluctuating temperatures and volume fractions facilitated by innovative empirical correlations.
- An increase in volume fractions led to agglomeration, which reduced the effectiveness of nanofluids in EOR. However, an increase in the inlet temperature resulted in an increase in the thermal conductivity of the nanofluid due to increased Brownian motion. This phenomenon helped to break up agglomerates and improved the oil recovery factor.
- The amplification of NF density due to the heightened temperature and VF fostered oil recovery by diminishing the mobility ratio and augmenting displacement efficiency.
- The increase in the volume fraction led to an increase in the viscosity of the nanofluid. In contrast, increasing the inlet temperature decreased oil viscosity, making it easier for extraction and increasing oil production.
- Notably, the simulation results were analyzed and compared using an NF and pure water. Adding the SiO${}_{2}$ NPs with a VF of 4% and an NF inlet temperature of 353.15 K resulted in 30% more oil production than water alone for flooding.
- A significant decrease in the ORF was observed when the VF was more than 4% and the inlet temperature exceeded 353.15 K.

Finally, this study is valuable to the field as it models the injection of nanofluids into a 3D porous medium, which can improve oil recovery. The developed mathematical model accurately accounts for changes in the properties of nanofluids, including the density, viscosity, and thermal conductivity, with variations in the temperature and volume fraction. The results are useful for engineering applications, particularly in optimizing the use of nanofluids for enhanced oil recovery. This study emphasizes the impact of aggregation on nanofluid effectiveness and shows how higher inlet temperatures can disperse agglomerates and enhance oil recovery.

## Figures and Tables

**Table 1:** Properties of the oil.

Property	Oil [50]	Water [51]	Unit
Density $\left(\rho_{o}\right)$	880	990	kg/m${}^{3}$
Viscosity $\left(\mu_{o}\right)$	$4.5\times 10^{-4}$	0.001	Pa.s
Specific heat capacity $\left(C_{o}\right)$	1670	4200	J/(kg·K)
TC coefficient $\left(k_{o}\right)$	0.13	0.6	W/(m·K)

**Table 2:** Properties of the NPs and porous media.

Property	SiO${}_{2}$ [52]	Reservoir [53]	Unit
Density (ρ)	3970	2714	kg/m${}^{3}$
Viscosity (μ)	0.0011	-	Pa·s
Specific heat capacity (C)	765	851	J/(kg·K)
TC coefficient (k)	40	2.2	W/(m·K)
Diameter of NPs	5	3	nm
Molecular weight	101.96	-	W/(m·K)

**Table 3:** PM properties.

Property	Value	Unit
Cross-section area (*A*)	$A=\pi^{2}h$	m${}^{2}$
Volume (*V*)	$V=\pi^{2}h$	m${}^{3}$
Height (*h*)	3	m
Radius (*r*)	1	m
Porosity (ε)	0.27	%
Permeability (K)	$1\times 10^{-9}$	m${}^{2}$
Initial NF saturation	0	%

**Table 4:** Boundary conditions.

The cylinder inlet surface	S${}_{o}$ = 0	**n.**$\rho_{nf}u_{nf}=0.01$ m/s	$T=T_{in}$
The rectangular prism outlet surface	$p_{o}=0$ Pa	$-n\cdot \rho_{o}u_{o}=\rho_{o}u_{o0}$	$-n\cdot k_{\mathrm{eff}}\nabla T=0$
All surfaces except the inlet and outlet		$-n\cdot \rho_{o}u_{o}=0$	$-n\cdot k_{\mathrm{eff}}\nabla T=0$
Initial condition	S${}_{o}$ = 1	$p_{nf}=0\;\mathrm{Pa}$	$T_{init}=293.15$ K

**Table 5:** Oil recovery factor at specific points (1 m, 0.5 m, 0.5 m, and 200 s) with different grids.

No.	No. of Elements	ORF
1	30,000	0.572
2	40,000	0.623
3	50,000	0.630
4	60,000	0.6445
5	70,000	0.6449
6	80,000	0.6451

**Table 6:** PM specifications.

Property	Value
Size (m${}^{3}$)	$4.836\times 10^{-15}$
Porosity (%)	0.25
Absolute permeability (m${}^{2}$)	$5.2\times 10^{-20}$

## Data Availability

The data presented in this study are available from the corresponding author upon reasonable request.

## Nomenclature

The following abbreviations are used in this manuscript:

**Greek Letters**	
α	Thermal diffusivity
ΔP	Pressure drop
ΔT	Temperature difference
ϵ	Porosity
$\kappa_{r,nf}$	Relative permeability of the NF
$\kappa_{r,o}$	Relative permeability of the oil
λ	Pore-size distribution index
μ	Dynamic viscosity
ρ	Density
θ	Contact angle
**Latin Letters**	
$B_{c}$	Square root of the permeability
*C*	NP concentration
$C_{p}$	Specific heat capacity
*D*	Diffusivity
$D_{c}$	Diffusion coefficient
*g*	Acceleration due to gravity
*h*	Film thickness
*K*	Absolute permeability
*L*	Characteristic length
*P*	Pressure
$P_{c}$	Capillary pressure
Pr	Prandtl number
Re	Reynolds number
$S_{e}$	Effective saturation
$S_{nf}$	NF saturation
$S_{o}$	Oil saturation
$S_{rnf}$	Residual saturation of the NF
$S_{ro}$	Oil residual saturation
*T*	Temperature
$T_{in}$	Inlet temperature of the NF
$T_{init}$	Initial temperature of the system
*t*	Time
*u*	Flow velocity
$u_{nf}$	Input velocity of the inlet fluid
VF	Volume Fraction
**Dimensionless Numbers**	
Bi	Biot number
Nu	Nusselt number
Pe	Peclet number
Pr	Prandtl number
Re	Reynolds number
Sc	Schmidt number
Sh	Sherwood number
